# Clay-Alginate Beads Loaded with Ionic Liquids: Potential Adsorbents for the Efficient Extraction of Oil from Produced Water

**DOI:** 10.3390/polym14204440

**Published:** 2022-10-20

**Authors:** Shehzad Liaqat, Taleb H. Ibrahim, Mustafa I. Khamis, Paul Nancarrow, Mohamed Yehia Abouleish

**Affiliations:** 1 Department of Chemical Engineering, College of Engineering, American University of Sharjah, Sharjah P.O. Box 26666, United Arab Emirates; 2 Department of Biology, Chemistry and Environmental Sciences, American University of Sharjah, Sharjah P.O. Box 26666, United Arab Emirates

**Keywords:** produced water, oil removal, clay-alginate-ILs beads, adsorption

## Abstract

Produced water (PW) generated from the petroleum industry, during the extraction of oil and gas, has harmful impacts on human health and aquatic life, due to its complex nature. Therefore, it is necessary to treat it before discharging it into the environment in order to avoid serious environmental concerns. In this research, oil adsorption from PW was investigated using clay-alginate beads loaded with ionic liquids (ILs), as the adsorbent material. The effects of several process parameters, such as the initial concentration of oil, contact time, pH, and temperature on the removal efficiency of the beads, were analyzed and optimized. Different characterization methods, such as the Fourier transform infrared spectrophotometer (FTIR), scanning electron microscopy (SEM), energy dispersive X-ray (EDX), and thermal gravimetric analysis (TGA), were used to investigate the surface morphology, the chemical bond structure and functional group, and the thermal stability of the ILs-based beads. The results revealed that the clay-alginate-ILs beads indicated a removal efficiency of 71.8% at the optimum conditions (600 ppm initial oil concentration, 70 min contact time, 10 pH, and at room temperature) with an adsorption capacity of 431 mg/g. The FTIR analysis confirmed the successful chemical bond interaction of the oil with the beads. The SEM analysis verified that the beads have a porous and rough surface, which is appropriate for the adsorption of oil onto the bead’s surface. The TGA analysis provides the thermal degradation profile for the clay-alginate-ILs. The beads used in the adsorption process were regenerated and used for up to four cycles.

## 1. Introduction

Produced water (PW) is the main wastewater stream caused by the petroleum industry throughout the extraction of the oil and gas industry. It consists of a wide variety of hydrocarbons in dissolved, dispersed, and free forms [1]. It comprises complex organic and inorganic compounds, such as dispersed and dissolved oil, greases, formation solids, heavy metals, waxes, scale products, salts, dissolved gases, and microorganisms [2]. In the literature, it is shown that the oil and gas industry is producing 250 million barrels of PW per day and more than 40% of the PW is being discharged into the environment, globally [3]. The discharging of untreated PW can cause serious environmental issues and lead to pollute the soil, underground, and surface water. These risks come due to the lack of the appropriate treatment technology, therefore multiple PW treatment technologies are being used [4]. These technologies can be divided into physical, chemical and biological methods [5,6,7], filtration [8], chemical oxidation [9,10], electrochemical oxidation [11], coagulation [12,13,14], and membrane separation [15,16,17]. Each technique has its restriction, such as a high cost, a low removal efficiency, and the generation of byproducts [18,19]. Adsorption is considered the utmost extensive and conventional purification method for the removal of organic, inorganic, and biological pollutants, which are soluble and insoluble in water [20,21,22,23,24,25]. Adsorption is an environmentally friendly, cost-effective, and economic method and can substitute the traditional PW treatment methods [26,27]. Moreover, the adsorption process has a wide range of applications and attractive removal efficiency with a simple design and operation. It can target specific pollutants easily and the absorbent can be recovered using different methods, such as desorbing, leaching, and thermal and biological processes [28].

Due to the environmental concerns, researchers are focusing on green and sustainable materials and technologies. Ionic liquids (ILs) received a lot of attention in different research fields during the last decade. ILs are molten salts with a melting point less than 100 °C, and comprise organic cations (e.g., imidazolium, pyridinium, pyrrolidinium, ammonium) with either organic or inorganic anions (e.g., bis (trifluoromethyl sulfonyl) imide, hexafluorophosphate, halides) [29,30,31]. Several synonyms are being used for ILs, such as ionic fluids, molten salts, fused salt, and liquid organic salts. The unique properties of ILs, such as the high thermal stability, ionic conductivity, dissolving ability, polarity, viscosity, and negligible vapor pressure, non-flammability, non-volatility, and hydrophobicity, make them a potential candidate for multiple applications [32]. Moreover, due to their design flexibility nature, ILs can be tailored for task-specific applications. The ILs cover a wide range of applications, such as separation processes, electrochemical processes, catalysis, thermal energy storage, heat transfer fluids, fuel cells, batteries, lubricants, and as additives, due to its attractive properties [33].

The literature shows that ILs and IL-based materials have a good potential for wastewater treatment and are being used for the removal of multiple pollutants from wastewater [34,35,36,37,38,39]. In this research, clay-alginate beads, loaded with ILs, were synthesized and successfully employed for the oil adsorption from PW, through the adsorption process. The Fourier transform infrared spectrophotometer (FTIR), scanning electron microscopy (SEM), energy dispersive X-ray (EDX), and thermal gravimetric analysis (TGA) were used to characterize the synthesized composite material. The effect of the different process parameters, such as the initial oil concentration, pH, contact time, and temperature, were studied. The adsorption kinetics and isotherms studies were also investigated by fitting the experimental data to different models.

## 2. Materials and Methods

### 2.1. Materials and Instrumentations

Heavy crude oil (HCO) was supplied by Abu Dhabi Oil company (ADNOC, Abu Dhabi, United Arab Emirates), United Arab Emirates, and the surfactant (ENDOR OCC9783, Dubai, United Arab Emirates) was provided by Suez, United Arab Emirates. The IL (1-Ethyl-3-Metyhlimidazolium acetate > 98%) was purchased from Iolitec, Heilbronn, Germany. This IL was selected, due to its attractive properties of a low melting point, viscosity, toxicity, and corrosiveness [40]. Potassium bromide (FTIR grade > 99%), and n-Hexane were obtained from Sigma Aldrich, Taufkirchen, Germany. Sodium alginate with a viscosity of 2000 centipoise (cp) was obtained from SFDCL, Mumbai, India. Raw clay was used during the bead’s preparation. Sodium hydroxide (NaOH, 99%), calcium chloride dihydrate, and hydrochloric acid (HCl, 37%) were obtained from Merck, Darmstadt, Germany. All other chemicals were of analytical grade and were used without any purification. The PW was prepared by adding HCO to the surfactant-water solution. The surfactant-water solution was prepared by mixing the surfactant and water with a 40:60 ratio. Distilled water was used during the whole of the experiment. The pH of the solutions were adjusted by either using 0.1 M HCl or 0.1 M NaOH.

A Water Still Aquatron (A4000D, Lane End, Buckinghamshire, United Kingdom) was used for the distilled water. The Stuart Vortex mixer (Cole-Parmer, Cambridgeshire, United Kingdom) was used for homogenous mixing. A centrifuge (HERMLE Labortechnik, Wehingen, Germany) was used to separate the oil and water layers. To perform the pH measurements, a pH meter (Oakton pH 510 series, EUTECH INSTRUMENTS, klang, Selangor, Malaysia) was used. A UV-Vis spectrophotometer (Thermo Scientific Evolution 220, Shanghai, China) was used to determine the concentration of oil in the PW. The FTIR (Perkin Elmer, Waltham, MA, USA) was used to analyze the presence of the functional groups and the chemical bond structure of the oil with the beads. To study the surface morphology of the beads, a SEM (TESCAN Vega 3, INCAx-act, Oxford instruments, Oxfordshire, United Kingdom) equipped with EDX was used. The thermal degradation of the beads was observed using the TGA (SHIMADZU DTG-60AH, Columbia, Maryland, United States of America).

### 2.2. Methodology

#### 2.2.1. Preparation of the Synthetic Produced Water

The synthetic PW was prepared by using HCO, surfactant, and distilled water. Firstly, the solution of the surfactant-water (40:60) was prepared by adding them together in a specific ratio of 40 mL surfactant and 60 mL of water and sonicated for 10 min. Then, the solution of different concentrations of PW was prepared by adding a specific amount of oil in the surfactant-water (40:60) solution and sonicated for 10 min, to prepare a homogenous emulsion of oil in water. 20 mg of HCO were added to the 100 mL solution of the surfactant-water, to prepare a 200-ppm solution of PW. Similarly, the different concentrations of PW, ranging from 100 to 700 ppm, were prepared by adding the respective amount of oil in the surfactant-water solution, which was used, for further experiments.

#### 2.2.2. Preparation of the Clay-Alginate-IL Beads

Sodium alginate, calcium chloride dihydrate, raw clay, IL, 1-ethyl-3-metyhlimidazolium acetate [EMIM][Ac], and distilled water were used for the bead’s preparation. First, 0.5 g of ILs were added to 25 mL of water to prepare the IL-water solution. Then, 0.5 g of raw clay was mixed with 0.5 g of sodium alginate to obtain the mixture in a powder form. Next, the powder was added slowly to the already prepared IL-water solution at 50 °C, using a heat stirrer (Stuart CB162, Cole-Parmer, Cambridgeshire, United Kingdom) to obtain an alginate-clay-ILs solution. Following 1 h of continuous stirring, a viscous solution of alginate-clay-IL was formed. This alginate-clay-ILs solution was added, drop by drop, to the 5% (*w*/*w*) solution of calcium chloride, using a magnetic stirrer (WiseStir MSH-20D, Wisd Laboratory Instruments, ZevenHuizen, The Netherlands). The uniform solid beads started to form as this viscous solution was added to the calcium chloride solution. The beads were stirred for 2 h in the calcium chloride solution for a better cross-linking and stability. Then, these beads were taken out of the calcium chloride solution and washed with distilled water for 15 min, to remove all of the calcium ions on the surface of the beads. Next, the beads were kept in the open air for 15 h to remove the moisture. Similarly, the clay-alginate beads without IL, were prepared. Then, both types of beads were tested for the removal of oil from the PW. It was found that the clay-alginate beads loaded with ILs are highly efficient, as compared to the clay-alginate beads without ILs. Therefore, the clay-alginate beads loaded with ILs were used for the removal of oil from the PW via the adsorption process, in this research.

#### 2.2.3. Adsorption Study

The beads were prepared by the immobilization of the ILs on a solid supporting surface, to remove the oil from the PW. Ten mg of the beads were added to a 10 mL solution of the PW and were placed in the shaker for 60 min, for a maximum mass transfer of the oil onto the beads. Once the beads were separated from the PW, the PW was analyzed using UV-visible spectroscopy at the wavelength of 275 nm, for the remaining oil. Equations (1) and (2) were used to calculate the adsorption capacity q_e_ (mg/g) and removal efficiency, respectively [41,42].

(1) $$q_{e}=(C_{i}-C_{f})\times \frac{V}{m}$$

(2) $$\%\;R=\left(\frac{C_{i}-C_{f}}{C_{f}}\right)\times 100$$

Here, C_i_ and C_f_ are the initial and final concentrations (mg/L) of the PW, respectively. V is the volume of the PW used, and m is the mass of the beads used.

#### 2.2.4. Process Optimization

To maximize the removal efficiency of the beads from the PW, the effect of the different parameters, such as pH, initial concentration of oil, contact time, and the temperature, were studied, to identify the optimum conditions. To study the effect of pH, different solutions of the PW with a pH ranging from 2 to 14 were prepared. The effect of the initial concentration of oil on the removal efficiency of the beads was studied by preparing a solution of different concentrations, ranging from 100 to 600 mg/L. The effect of contact time, ranging from 10 to 90 min, on the removal efficiency of the beads was also studied. The effect of the temperature on the removal efficiency of the beads was also examined. The adsorption kinetics and isotherm studies were performed for the adsorption of oil through the beads.

#### 2.2.5. Characterization of the Materials Used in the Adsorption Study

Different characterization techniques were used to confirm the adsorption of oil by the beads. The FTIR analysis, ranging from 4000 to 500 cm^−1^, was used to identify the functional groups and the chemical bond structures and interaction of the oil with the beads. The SEM-EDX analysis was used to study the surface morphology of the beads. The thermal degradation of the beads was observed by the TGA ranging from 50 to 500 °C with a heating rate of 10 °C/min in a nitrogen environment. The UV-visible spectrophotometry was used to determine the concentration of oil in the PW.

#### 2.2.6. Regeneration Study of the Clay-Alginate-IL Beads

The removal of oil from the PW was completed by adding 10 mg of the beads in a 10 mL solution of the PW. To use the same beads for the second cycle, the regeneration of these beads was carried out by washing the beads with ethyl acetate. For this purpose, 10 mg of the beads was added to the ethyl acetate at 100 rpm for 30 min. Then, the beads were washed with distilled water for 10 min and were used for the second cycle after drying. The same regeneration procedure was performed to use the beads for the 3rd and 4th cycles.

## 3. Results and Discussion

### 3.1. FTIR Analysis of the Materials Used in the Adsorption Process

**Figure 1:**
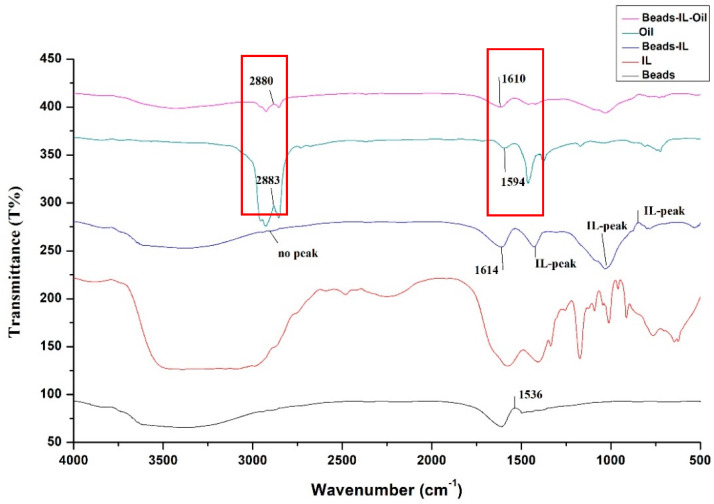
FTIR analysis before and after the adsorption of oil onto the surface of the beads.

To study the chemical bond interaction between the oil and the clay-alginate-ILs beads, the FTIR analysis was performed. The spectra of oil, clay-alginate-ILs beads, and clay-alginate-ILs-oil beads were recorded in the range of 4000 to 500 cm^−1^. The experimental study revealed that the beads have a good ability to extract oil from the PW with an adsorption capacity of 431 mg/g. Figure 1 represents the FTIR analysis before and after the adsorption of the oil onto the surface of the beads. The peaks in the single bond region (2500–4000 cm^−1^) are associated with the (C-H, N-H, O-H) bonds. The peaks in the double bond region (1500–2000 cm^−1^) are due to the presence of the (C=C, and C=O) bonds. It can be concluded from comparing the spectra of the beads, IL, and beads-IL that IL-peaks are appearing in the bead-IL spectra upon modification which confirms the successful loading of the IL into the beads. Inspection of Figure 1 also reveals that there is no peak for the clay-alginate-ILs beads in the region 2800–3000 cm^−1^ before the use of the beads for the PW treatment. However, after applying the beads for adsorption, a peak at 2883 cm^−1^ appeared which represents the adsorption of the oil on the active sites of the beads. A peak shifting was also noticed from 1614 to 1610, demonstrating the stretching and vibration of the molecules, due to their interaction. Similar trends were found in the literature for the alginate beads loaded with ILs for the decontamination of pollutants from the aqueous phase [43].

### 3.2. TGA of the Clay-Alginate-ILs Beads and the Na-Alginate Used in the Adsorption Process

**Figure 2:**
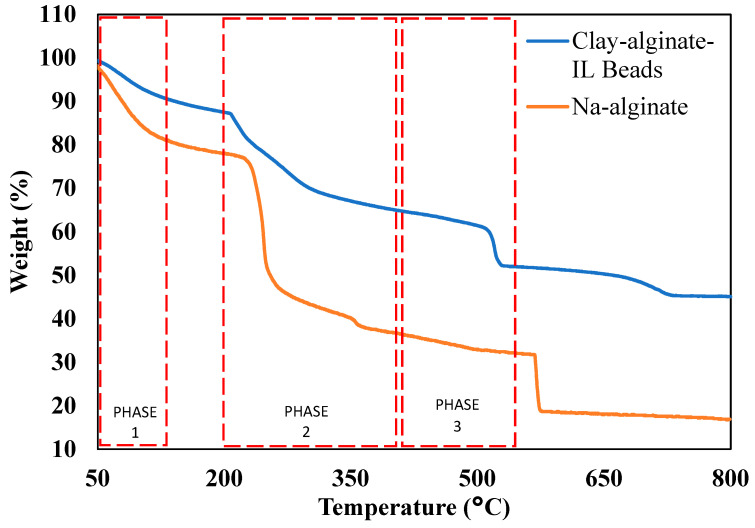
TGA of the clay-alginate-ILs beads and Na-alginate used in the adsorption process.

The thermal degradation profile of the beads and Na-alginate were recorded to study their behavior and thermal stability, using the TGA instrument, at a temperature range of 50 to 800 °C with a heating rate of 10 °C/min in an N_2_ environment. Figure 2 represents the TGA of the beads and Na-alginate.

Figure 2 demonstrates the loss in weight with the increment in temperature. The TGA profile showed that thermal degradation of both the beads and the Na-alginate is increasing with the temperature. There are three phases for the degradation of the beads and Na-alginate [44]. The first phase lies between 50 to 120 °C, where a small amount of mass was decreased, which represents the removal of the moisture content [44]. Once the temperature reached 200°C, an approximate 10% weight loss has been observed for the clay-alginate-ILs beads. The second phase can be observed between 200 and 400 °C, which represents the pyrolysis of the beads where a significant amount of weight loss can be observed due to degradation of the ILs and sodium alginate [44]. Once the temperature reached 500 °C, an approximate 40% weight of the clay-alginate-ILs beads has been decreased, whereas approximately 70% of weight of the Na-alginate has been lost. This represents that the IL has improved the thermal stability of the beads [45]. The third phase is called the passive region, which lies between 400 to 550 °C where the degradation of the component is occurring. Once the temperature reached 800 °C, about 50% of the weight of the clay-alginate-IL beads has been lost and it becomes constant, which represents that only clay is left behind and the ILs and sodium-alginate already have been degraded. A similar degradation profile was reported in the literature for Na-alginatse beads [45,46].

### 3.3. SEM-EDX Analysis of the Clay-Alginate-ILs Beads

**Figure 3:**
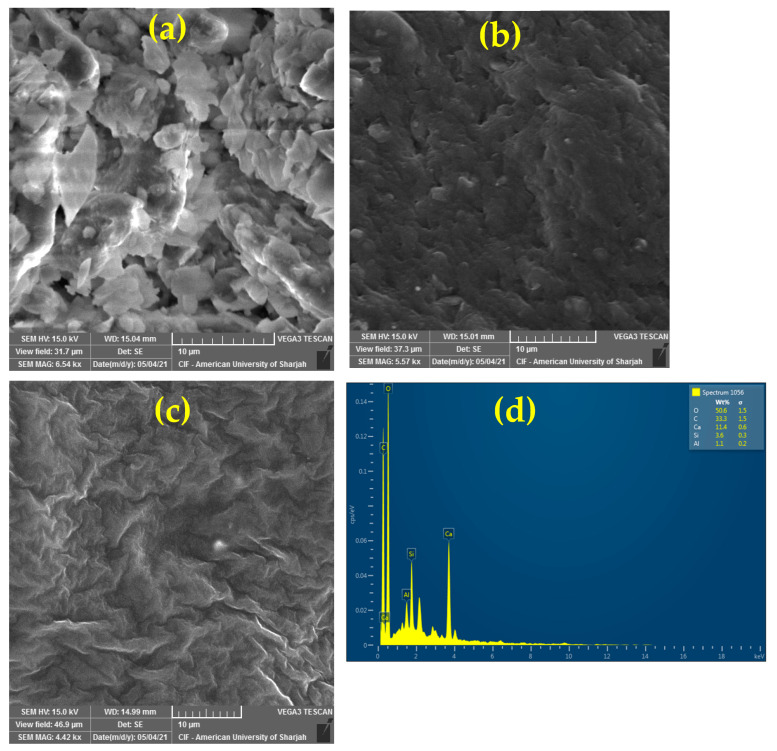
SEM images of the clay-alginate-ILs beads with different magnifications (**a**) 31.7 µm, (**b**) 37.3 µm, (**c**) 46.9 µm (**d**) EDX analysis of the clay-alginate-IL beads.

To study the surface morphology and texture of the beads, a SEM-EDX analysis was performed. Figure 3a–c presents the SEM of the clay-Na alginate-ILs beads with different magnifications, and Figure 3d displays the elemental analysis of the beads.

Figure 3 shows that the ILs-based beads have a porous-rough surface which is helpful for the adsorption of oil onto the surface of the beads [47,48]. In the literature, it has been reported that the Na-alginate beads have a porous surface, which is efficient for the adsorption of oil onto the beads [49]. The EDX analysis showed the elemental composition of the major elements present in the beads, such as oxygen (O), calcium (Ca), and silicon (Si).

### 3.4. Effect of the pH of the PW Solution on the Adsorption Capacity of the Beads

**Figure 4:**
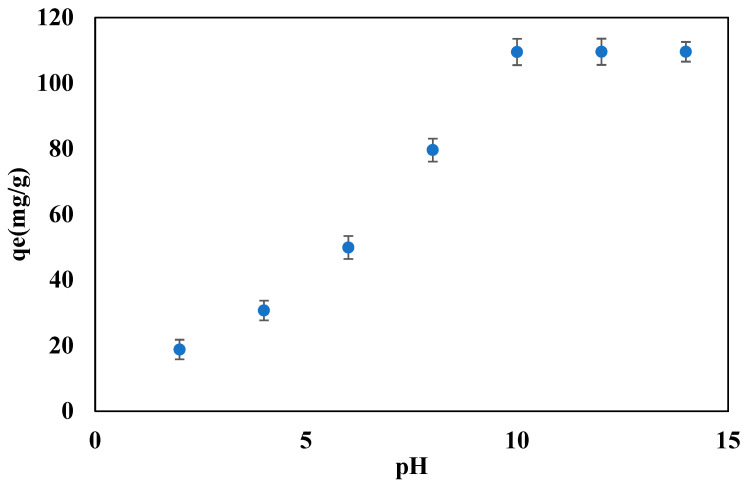
Effect of the pH on the adsorption capacity of the beads at 60 min contact time, 200 ppm initial oil concentration, 10 mg of beads.

To study the effect of the pH on the adsorption capacity of the beads, the PW solutions at a different pH, ranging from 2 to 14, were prepared. The adsorption capacity at each pH was calculated at an initial concentration of oil of 200 ppm, contact time 60 min, beads dosage of 10 mg, and 10 mL PW at room temperature and pressure. Figure 4 presents the effect of the pH on the adsorption capacity of the beads, where the adsorption capacity increased from 18.8 to 109.5 mg/g as the pH increased from 2 to 10.

The adsorption capacity became constant at the value of 109.5 mg/g when the pH changed from 10 to 14. A pH 10 was considered the optimal value for further experiments. Figure 4, demonstrates that the adsorption capacity is low in the acidic region, compared to the basic region. Inspection of Figure 4 reveals that the adsorption capacity is low in the acidic region and increases with the increasing pH. The adsorption capacity at a low pH could be attributed to a larger mass transfer resistance, which minimizes the mass transfer between the oil and the beads. In the basic region, the mass transfer resistance decreases, and the driving force for the mass transfer between the oil and the beads increases, which leads to the observed increase of the adsorption capacity of the beads. A similar trend was reported in the literature for the adsorption of methylene blue dye [50], which was attributed to an increase in the negative charge of the surface leading to a larger coulombic attraction between the cationic dye and the surface. In the present study, however, the PW contains a variety of organic pollutants that their molecular structure is expected to undergo a slight change at a high pH. Hence, the observed increase in the adsorption capacity at a high pH is most likely to be due to the surface structure changes of the beads, which leads to the generation of new active sites for adsorption.

### 3.5. Effect of the Oil Initial Concentration on the Adsorption Capacity of the Beads

The initial concentration of the oil in the PW solution can affect the adsorption capacity of the beads [51]. To study the effect of the initial oil concentration on the adsorption capacity of the solid-supported ILs beads, the PW solutions of different initial oil concentrations, ranging from 100 to 600 ppm, were prepared. The adsorption capacity of the ILs-based beads was studied for each solution of the PW at pH 10, 60 min contact time, 10 mg beads dosage, and 10 mL PW at room temperature and pressure.

**Figure 5:**
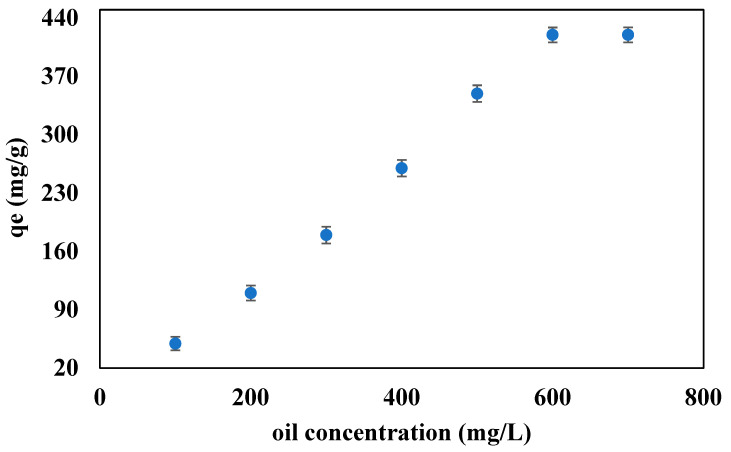
Effect of the initial oil concentration on the adsorption capacity of the beads at 60 min contact time, pH 10, and 10 mg of beads.

Figure 5 displays the effect of the initial concentration of oil in the PW on the adsorption capacity of the ILs-based beads. Figure 5 showed that the adsorption capacity of the beads followed an increasing trend with the initial concentration of oil in the PW solution. This increase in the adsorption capacity is due to the transfer of more oil towards the beads at a higher initial concentration, as compared to the small initial concentration. At a higher initial concentration, the driving force for the mass transfer is higher and the mass transfer resistance is lower, which causes the transfer of more oil towards the active site of the beads. An initial concentration of 600 ppm with a maximum adsorption capacity of 420 mg/g was taken as the optimum concentration for further experiments. A similar trend was reported in the literature for the alginate beads for the removal of pollutants from the aqueous phase [49].

### 3.6. Effect of the Contact Time on the Adsorption Capacity of the Beads

**Figure 6:**
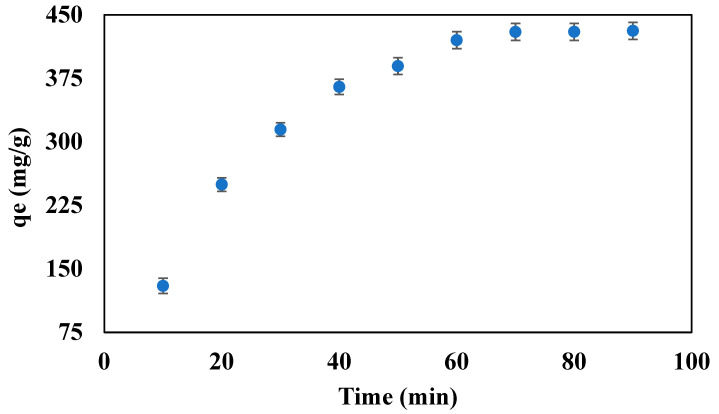
Effect of the contact time on the adsorption capacity of the beads at 600 ppm initial oil concentration, pH 10, and 10 mg beads dosage.

To study the effect of the contact time on the adsorption capacity, the beads were tested for different time intervals, ranging from 10 to 90 min, at pH 10, 600 ppm initial oil concentration, 10 mg beads dosage, and 10 mL PW at room temperature and pressure. Figure 6 presents the effect of the contact time on the adsorption capacity of the sodium-alginate-ILs-based beads. Figure 6 demonstrates that the adsorption capacity increased with time and became constant (429.8 mg/g) after 70 min. This increase in the adsorption capacity is due to the transfer of a greater number of molecules of oil towards the sodium-alginate-ILs-based beads at a higher contact time compared to less contact time. The adsorption capacity almost became constant at 429.8 mg/g after 70 min because the beads were saturated, and no mass transfer occurred between the oil and beads. A contact time of 70 min with a maximum adsorption capacity of 429.8 mg/g, was taken as the optimal time for further experiments. The literature also reported, as the contact time was increased, the adsorption capacity was also increased for the alginate beads [49].

### 3.7. Effect of the Temperature on the Adsorption Capacity of the Beads

Temperature can affect the adsorption capacity of the solid-supported ILs beads during the adsorption of oil onto the solid beads. Therefore, the effect of temperature on the adsorption capacity of the ILs beads was studied.

**Figure 7:**
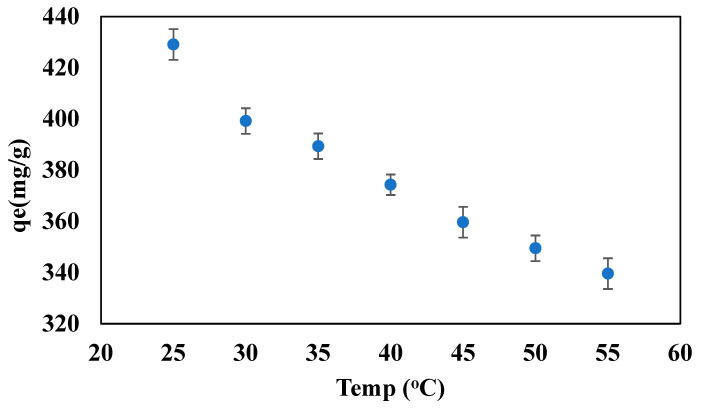
Effect of the temperature on the adsorption capacity of the beads at 600 ppm initial oil concentration, pH 10, 10 mg beads dosage, and 70 min contact time.

The beads were tested at different temperatures, ranging from 25 to 55 °C, at the following optimum conditions, pH 10, 600 ppm initial oil concentration, 25 mg beads dosage, and 10 mL PW at room pressure. Figure 7 presents the temperature effect on the adsorption capacity of the beads. Figure 7 showed that the adsorption capacity has an inverse relation with the temperature, where the adsorption capacity decreased from 429.1 to 339.6 mg/g when the temperature increased from 25 to 55 °C. This decrease in the adsorption capacity indicates that the adsorption process is, as expected, exothermic in nature with negative enthalpy changes in the adsorption. Hence, increasing the temperature will result in decreasing the adsorption capacity, due to the gain of sufficient kinetic energy to overcome the weak bonds of the physical adsorption.

As a result, the room temperature was selected as an optimal temperature, which is also economical in terms of energy and cost. A similar trend was reported in the literature for the adsorption of heavy metals from industrial wastewater on the clay-based adsorbent [52].

### 3.8. Regeneration Study of the Beads Used in the Adsorption Process

**Figure 8:**
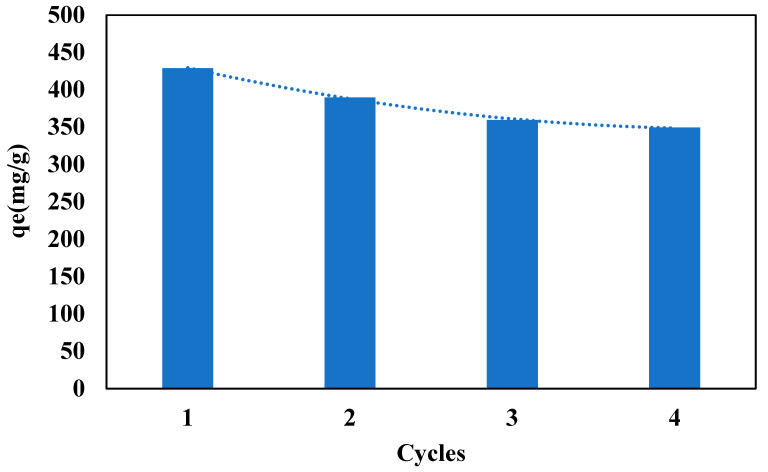
Regeneration study of the clay-alginate-ILs beads.

The regeneration of the beads is important from an economical point of view. The beads will be graded more efficiently if they can be reused for multiple cycles after regeneration. Therefore, a regeneration study was also performed to regenerate and use the tested beads for further cycles. The regeneration of the beads was achieved by keeping the beads in ethyl acetate for 30 min with continuous shaking of 100 rpm, so that, the oil can be removed from the saturated beads, and as a result, more active sites would be available for further cycles. Then, the beads were washed with distilled water and dried before using them for the next cycles. A similar procedure was adopted for the 3rd and 4th cycles. Figure 8 presents the adsorption capacity for the different cycles. The experimental results showed that the beads were efficient to use up to the 4th cycle with an 18.57% decrease in the adsorption capacity.

### 3.9. Adsorption Kinetics for the Adsorption of Oil onto the Clay-Alginate-ILs Beads’ Surface

Two kinetic models, a pseudo-first order and a pseudo-second order, were applied to the experimental data to investigate the kinetics of the oil adsorption onto the Na-alginate beads. Equations (3) and (4) were used to determine the kinetics parameters for the pseudo-first-order and the pseudo-second-order, respectively [53]. The rate constants of the pseudo-first order and pseudo-second orders are k_1_ (min^−1^) and k_2_ (g mg^−1^ min^−1^), respectively.

(3) $$\ln\left(q_{e}-q_{t}\right)={\mathrm{lnq}}_{e}-k_{1}t$$

(4) $$\frac{t}{q_{t}}=\frac{1}{k_{2}q_{e}^{2}}+\frac{1}{q_{e}}t$$

**Figure 9:**
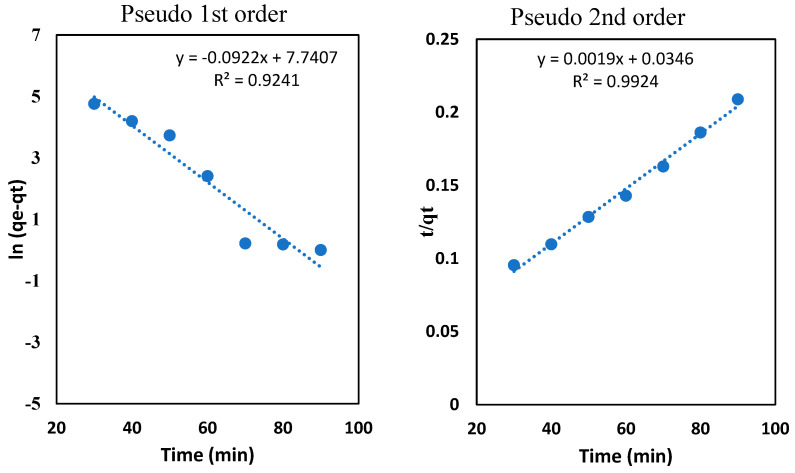
Adsorption kinetics study for the pseudo first and second orders.

Figure 9 represents the plot of [t vs. ln(q_e_−q_t_)] and [tv vs. t/q_t_] for the pseudo-first order and the pseudo-second order, respectively. Table 1 represents the kinetic parameters for both models. The regression coefficients for the pseudo-first and pseudo-second order are *R^2^=* 0.92 and *R*^2^*=* 0.99, respectively, which showed that the adsorption of oil into the beads is best described by the pseudo-second order. The adsorption capacity was also determined graphically with a maximum value of 625 mg/g, and with a rate constant of 7 × 10^−5^ g/mg min^−1^. The literature also showed that the adsorption kinetics of the oil extraction from the PW, using olive leaves [53], pomegranate peel [54], and eggplant peel powder [55] follows the pseudo-second order rate law. The pseudo-second order indicates that the chemisorption took place during the adsorption process. Figure 9 represents the plot of [t vs ln(q_e_ − q_t_)] and [tv vs t/q_t_] for the pseudo-first order and pseudo-second order, respectively. Table 1 represents the kinetic parameters for both models.

### 3.10. Adsorption in a Pseudo-Second Order Isotherm

Two isotherm models, Langmuir and Freundlich, were applied to the experimental data to study the adsorption isotherm of oil onto the beads, at room temperature. Equations (5) and (6) were used for these models, respectively [53]. where C_e_ (mg/L) represents the remaining oil in the aqueous phase at equilibrium, *q_m_* (mg/g) represents the maximum adsorption capacity of the beads, and b (L mg^−1^), shows the Langmuir adsorption constant, which is related to the free energy, k_f_ (L mg^−1^) is the equilibrium Freundlich constant and n represents the experiential constant and can be calculated by plotting ln q_e_ against ln C_e_.

(5) $$\frac{1}{q_{e}}=\frac{1}{{\mathrm{bC}}_{e}q_{\max}}+\frac{1}{q_{\max}}$$

(6) $$\ln q_{e}=\ln k_{f}+\frac{1}{n}\ln C_{e}$$

**Figure 10:**
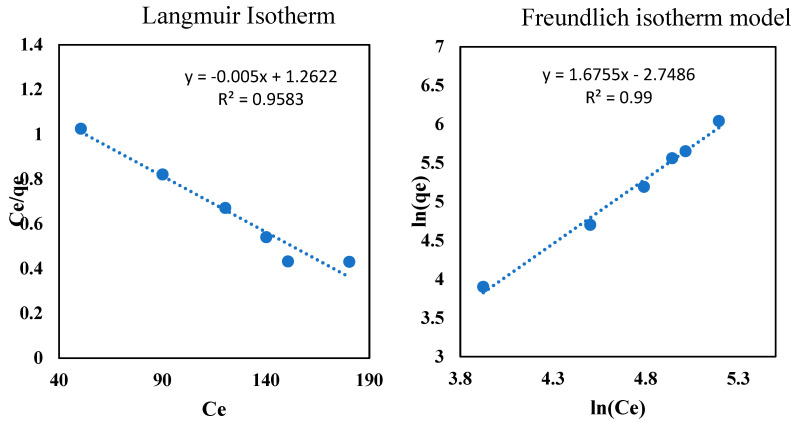
Adsorption isotherms study of the oil onto the clay-alginate-ILs beads’ surface.

Table 2 presents the calculated isotherm parameters. Figure 10 presents the Langmuir and Freundlich isotherm plots. The regression coefficients for the Langmuir model and Freundlich model are *R*^2^ *=* 0.998 and *R*^2^ *=* 0.990, respectively, which showed that the experimental data of the oil adsorption onto the beads was equally fit in both isotherms with the existing experimental uncertainty of the Langmuir isotherm model. The Langmuir model assumes the formation of a monolayer, while the Freundlich isotherm model removes this assumption. The literature also confirms that the extraction of the oil from the PW using kiwi peels [55], and the heavy metals removal using activated carbon, silica and silica activated carbon composite [56], follows the similar model. Inspection of Table 2 reveals that the q_max_ obtained from Langmuir model is 243.9 mg/g, which is significantly lower than that obtained from the kinetic model. This could be attributed to the discrepancy with the fitting equations, since q_max_ obtained from the plotting of q_e_ vs C_e_ is approaching the one obtained from the kinetic model.

### 3.11. Comparison with the Literature Data

Table 3 represents the adsorption capacity of the different adsorbents used for the PW treatment. Inspection of this table reveals that our adsorbents have a better adsorption capacity than most of those listed in the table.

## 4. Conclusions

Clay-alginate-ILs beads were synthesized and employed for the oil removal from the PW through the adsorption processes. During the adsorption process parameter optimization, it was examined that the adsorption capacity of the beads is affected by the pH of the PW, the initial oil concentration in the PW, contact time, and temperature. The adsorption capacity followed an increasing trend with the initial oil concentration, pH of the PW, and contact time, while it decreased with the temperature. The adsorption capacity of 431 mg/g was achieved with the clay-alginate-ILs beads at the optimum conditions (600 ppm initial oil concentration, 70 min contact time, pH 10, and at room temperature). Different characterization techniques, such as the FTIR, SEM-EDX, and TGA were applied to investigate the chemical bond interaction and functional groups, surface morphology, and thermal stability of the used materials. The FTIR analysis validated the successful chemical bonding between the oil and the beads. The SEM analysis demonstrated that the clay-alginate-ILs beads have a porous and rough surface. which is appropriate for the adsorption of oil onto the bead’s surface. The TGA analysis showed the thermal degradation profile of the clay-alginate-ILs beads, which indicated the weight loss with the temperature. Moreover, the clay-alginate-ILs beads utilized in the adsorption process were regenerated and used up to the 4th cycle.

## Figures and Tables

**Table 1:** Adsorption kinetics parameters of the oil into the clay-alginate-IL beads.

Kinetic Models	Parameters	Values
Pseudo first order	*k*_1_ (min^−1^)	0.0922
	*R* ^2^	0.9241
Pseudo second order	*k*_2_ (g/mg min^−1^)	1.6 × 10^−3^
	*R* ^2^	0.9970

**Table 2:** Adsorption isotherm parameters of the oil adsorption into the beads.

Isotherm Model	Parameters	Values (25 °C)
Langmuir model	b (L/mg)	0.00529
	q_max_ (mg/g)	243.9
	*R* ^2^	0.998
Freundlich model	K_f_ (mg/g) (L/mg)^1/n^	0.2239
	n	0.5968
	*R* ^2^	0.99

**Table 3:** Comparison of the different adsorbents used for the removal of oil from the PW.

Adsorbents	Adsorption Capacity (mg/g)	Reference
Kiwi peels	-	[41]
Walnut shell	-	[57]
Date pits	-	[57]
Graphene	100	[58]
Graphene magnetite	80	[58]
Olive leaves	143	[53]
Pomegranate peels	555	[54]
Multiwalled carbon nanotubes and their derivates	-	[59]
Organo-clay	67	[59]
Modified bentonite	49	[59]
Eggplant peels	834	[55]
Iron oxide nano adsorbents	-	[60]
Clay-alginate -IL beads	244	This work

## Data Availability

The data presented in this study are available on request from the corresponding author.
